# Supplementary material for: Identification of FGF13 as a Potential Biomarker and Target for Diagnosis of Impaired Glucose Tolerance
Source: Int J Mol Sci. 2023 Jan 16;24(2):1807. doi: 10.3390/ijms24021807 (PMC9867186; doi:10.3390/ijms24021807)
Supplement: Supplementary file 1 [file ijms-24-01807-s001.zip › ijms-2127500-supplementary.pdf]

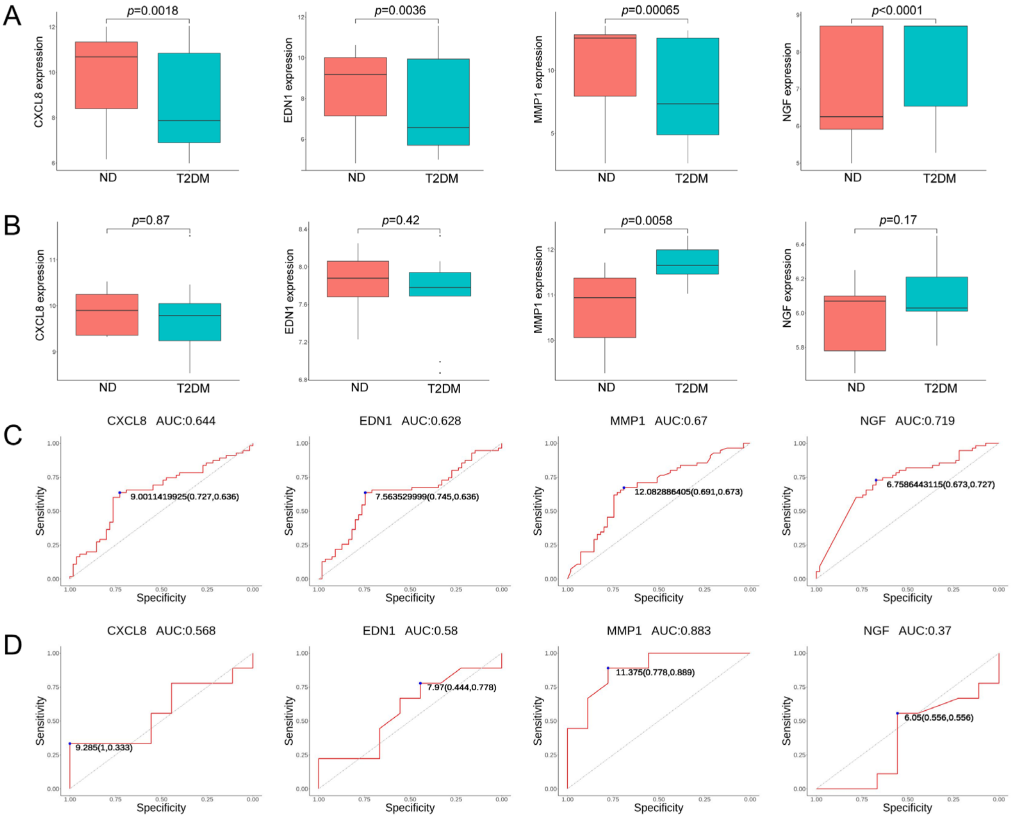

Figure S1. Validation of hub genes. (A, B) The expression of hub genes including CXCL8, EDN1, MMP1, and NGF was detected in GSE76896 and GSE38642. (C, D) ROC curve of the selected genes in GSE76896 and GSE38642.

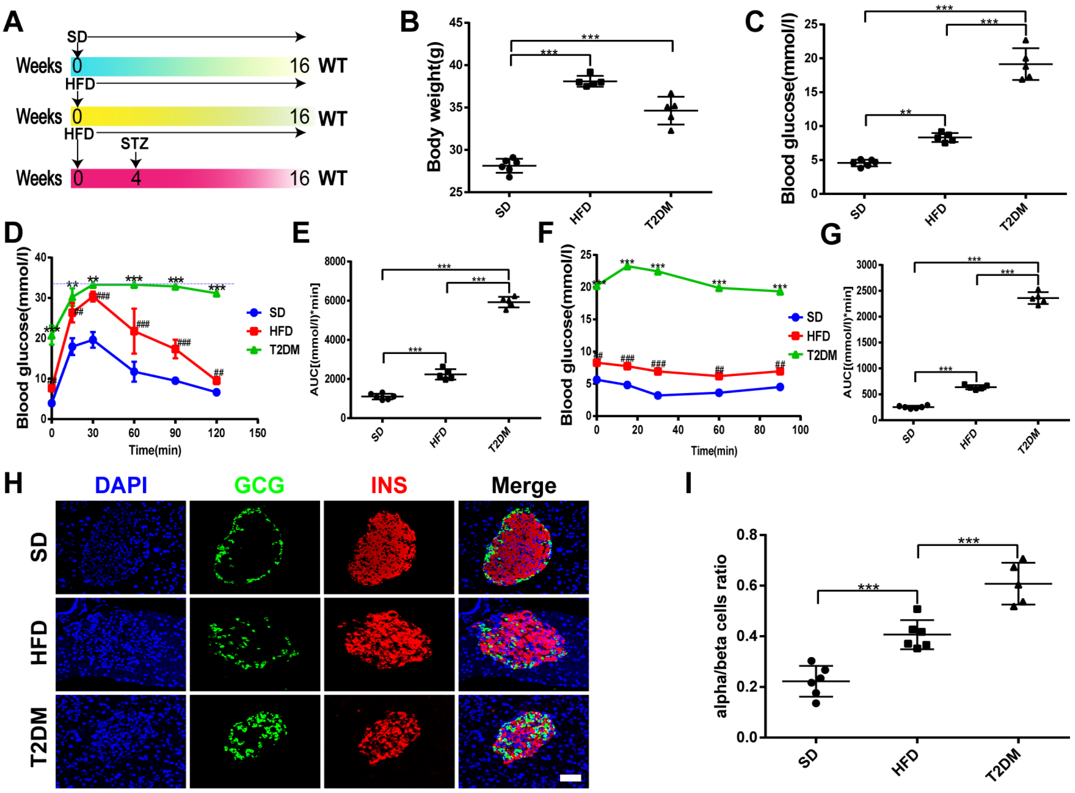

Figure S2. The construction of pancreatic islet models in mice with pre-diabetes and T2DM. (A) Experimental schedule. (B) Body weight. (C) Fasting blood glucose levels. (D, E) IPGTT was performed at 16 weeks (D), which were fasted for 12 h, and the AUC of the IPGTT (E). (F, G) IPITT was performed at 16 weeks (F), which was fasted for 12 h, and the AUC of IPITT (G). (H, I) Representative double-stained images of insulin and glucagon in pancreatic islets (H) and assessment of the pancreatic α/β-cell ratio (I). Scale bars = 20 μm. Values were expressed as means ± SD (*n* =5 or *n*=6). $^{\#\#}p < 0.01$ and $^{\#\#\#}p < 0.001$ vs. SD group; $^{**}p < 0.01$ and $^{***}p < 0.001$ vs. HFD group for (D, F). $^{**}p < 0.01$ and $^{***}p < 0.001$ for (B, C, E, G, I).
